# Notes on Preparations for the Sick

**Published:** 1906-09

**Authors:** 


### Notes on Preparations for the Sick.

**Validol.**—With reference to our note on this drug in the last number of the *Journal* (page 184), we are informed by Messrs. Widenmann & Co. (33 Lime Street, London) that the promoters of the drug, Messrs. Zimmer & Co., have no wish that its composition should remain a secret. They enclose some literary notes, from which we find that Validol is menthol (peppermint camphor, or rather menthol ester with 30 per cent. of free menthol) in chemical combination with valerianic acid; we are informed also that its sale is attaining very large proportions, thus showing that it is a much-appreciated combination which is proving itself useful in a great variety of neurasthenic conditions, and that its utility is not limited to the treatment of sea-sickness.

**Castor Oil Powder:** Risiccol-Siccolum-Ricini (Demuth).—Rudolph Demuth, 61 Mark Lane, London, E.C.—This powder, devoid of all taste and smell, contains about 50 per cent. of castor oil, and is a palatable and reliable aperient. It is a dry, slightly gritty powder, which can easily be mixed with water, tea, cocoa, milk, broth, porridge, &c., making an efficient but tasteless dose having all the therapeutic properties of castor oil. The excipient is said to be burnt magnesia, and it is very astonishing that the disagreeable qualities of the oil should have been absolutely abolished. The powder does not dissolve in water, but forms a fairly stable emulsion, from which the oil does not visibly float on the top even after standing for some time. The dose varies from one teaspoonful to a tablespoonful, but its action is somewhat delayed in comparison with the pure oil. This powder should be most useful in the treatment of habitual constipation, as also for children. Other "Siccol" preparations are made containing creosote, sandal oil, cod liver oil and oil of male fern.

**Salit.**—E. W. Blasius, 127 Fenchurch Street, London, E.C.—Salitum purum, prepared by the German firm, Von Heyden, Radebeul, Dresden, is another of the now numerous external applications for rheumatic troubles. The painful part should be cleansed with water, soap and spirit, then thoroughly dried and rubbed twice a day with one or two teaspoonfuls of Salit diluted with an equal quantity of olive oil. It has no disagreeable smell, it causes little cutaneous irritation, but it has been found to give speedy relief in cases of muscular rheumatism, lumbago, acute neuralgia, sciatica, articular rheumatism, teno-synovitis, and rheumatic pleurisy. We cannot have too many remedies for troubles of this kind, and it is convenient to be able to interchange from time to time if any one of them appears to be inefficient.

---

**Cocoids**: Diastase.—OPPENHEIMER, SON & CO., London.—These contain five grains of diastase in a chocolate basis in a palatable and convenient form for administration to those who dislike or decline to take other varieties of preparations containing the diastasic properties of the malt extracts. These Cocoids are prepared from a new diastase, which has a strength equivalent to 170 degrees Lintner: they should be a very reliable and convenient form for administration of a useful drug.

---

**Tabloid Mistura Alba.**—BURROUGHS, WELLCOME & CO., London.—Each contains: Magnesium sulphate gr. 15, magnesium carbonate gr. $2\frac{1}{2}$, oil of peppermint min. $\frac{1}{32}$ Tabloid Mistura Alba contains exact amounts of active ingredients, and presents a convenient and reliable means of administering this efficient stomachic and carminative combination. From one to eight may be powdered and taken in water for a dose. These tabloids are a very portable and convenient form for the easy preparation of a combination which has had a long-lived reputation, and which seems likely to take a new lease in tabloid form.

---

**Byno-phosphates.**—ALLEN & HANBURY, London, E.C.—This tonic food is a combination of liquid malt extract with the usual ingredients of the ordinary chemical food, viz. the phosphates of iron, lime, potash and soda. It forms a nearly neutral solution, and the Bynin element should assist in the digestion of starchy foods. We have found the Bynin group of preparations to be of great service. Patients take them and ask for more; accordingly they may be persuaded to continue them long enough to be of real service in removing long-standing debility, due to many and varied causes.

---

**New Skin**—DOUGLAS MANUFACTURING CO. (J. E. GARRATT), 124 Southwark Street, London, S.E.—This waterproof liquid court plaster appears to have decided advantages over the simple collodion. It has positive antiseptic qualities, and when it becomes dry it effectively covers the wound, preventing further contamination. Being transparent when dry, it allows continued examination of a wound without exposure, and it adheres so firmly that it can be washed freely with soap and water without becoming detached. A coating on the feet will prevent soreness from shoes, it will protect the hands of the surgeon from contamination by fœtid wounds or *post-mortem* examinations, and photographers can with it protect the nails from staining by silver salts. We are not informed what its exact composition may be, but it appears to be a solution of rubber, which obviously will serve very many useful purposes for the cure of surface wounds and for the protection of the sound skin.

---

**Evian:** Source Cachat.—INGRAM & ROYLE, London.—This saline table water has attained a great popularity, there being an annual sale of seven millions of bottles. It was first discovered and analysed in 1789. It is well aerated and agreeable to drink; administered methodically it is a powerful and harmless diuretic, so that "in a word, it disintoxicates the organism." It is obvious that it should be most useful in cases of chronic gout with cardiac and urinary complications. It was described by Jules Simon as "the best of all table waters."

---

**Tabloid Laxative Fruit Pastilles.**—BURROUGHS, WELLCOME & CO., London.—These pastilles present an efficient laxative in an exceptionally agreeable form, and possess distinct advantages over other aperient preparations. Each pastille contains five grains of extract of senna fruit in a suitable and pleasantly flavoured basis. The extract of senna fruit produces a gentle laxative effect without the griping and other discomfort associated with the administration of preparations of senna leaves. These Laxative Fruit Pastilles form an acceptable aperient, well suited to the needs of children and invalids when drastic purgatives are undesirable. As the aperient principle is excreted by the mammary gland, Tabloid Laxative Fruit may be given to a nursing mother in order that its effect may be exercised upon the infant.

---

**Tabloid Menthol and Eucalyptus Pastilles.**—BURROUGHS, WELLCOME & CO., London.—Each contains: Menthol gr. $\frac{1}{20}$, oil of eucalyptus min. $\frac{1}{2}$. An excellent combination for the treatment of infective, inflammatory, ulcerated or catarrhal conditions of the mouth and throat. The pastilles present a pleasant and efficient means of treatment. They ensure prolonged application to the affected parts, and of stimulating, anæsthetic, antiseptic and deodorant principles in suitable solution. They relieve hoarseness and throat pain, and strengthen the voice.

---

**Alypin.**—THE BAYER CO., LTD., London, E.C.—This new local anæsthetic, which is manufactured by the Bayer Co., possesses certain advantages over cocaine. It is much more easily sterilised: repeated boiling for a short time will not decompose it. It causes less dilatation of the pupil than cocaine, and should therefore be useful in some cases of glaucoma. Repeated instillation is said not to cause any opacity of the corneal epithelium. For these reasons it is perhaps a rather safer local sedative for patients to use occasionally. With cocaine there is always a risk of abrasion of the cornea and infection owing to the cocaine not being sterile. For ophthalmic operations its very slight toxic action presents no very special advantage over cocaine, and the fact that it causes rather more injection of the conjunctiva is a slight disadvantage, but admixture with adrenalin prevents this injection. It certainly is a very good local anæsthetic.

